# 6-Meth­oxy-2-[(*E*)-phenyl­imino­meth­yl]phenol

**DOI:** 10.1107/S1600536811009135

**Published:** 2011-03-15

**Authors:** Yu-Ye Yu

**Affiliations:** a Jinhua College of Vocation and Technology, Jinhua, Zhejiang 321017, People’s Republic of China

## Abstract

The title compound, C_14_H_13_NO_2_, was obtained by the condensation reaction of *o*-vanillin and aniline in ethanol. The mol­ecule adopts the phenol–imine tautomeric form and an *E* conformation with respect to the azomethine C=N bond. The dihedral angle between the aromatic rings is 30.57 (10)°. In the crystal, mol­ecules are linked by inter­molecular C—H⋯O hydrogen bonds into zigzag chains parallel to the *b* axis.

## Related literature

For related metal complexes with Schiff base ligands derived from *o*-vanillin and aniline, see: Li *et al.* (2008 ▶); Liu *et al.* (2009 ▶); Xian *et al.* (2008 ▶); Zhao *et al.* (2007 ▶). For the syntheses and anti­bacterial activities of rare earth complexes with Schiff base ligands derived from o-vanillin and adamantane­amine, see: Zhao *et al.* (2005 ▶).
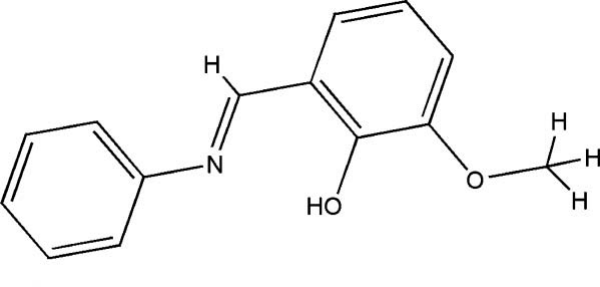


## Experimental

### 

#### Crystal data

C_14_H_13_NO_2_
*M* *_r_* = 227.25
Orthorhombic, 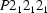

*a* = 6.0882 (4) Å
*b* = 9.1862 (5) Å
*c* = 21.0800 (12) Å
*V* = 1178.95 (12) Å^3^
*Z* = 4
Mo *K*α radiation
μ = 0.09 mm^−1^
*T* = 296 K
0.33 × 0.22 × 0.18 mm

#### Data collection

Bruker APEXII area-detector diffractometer
Absorption correction: multi-scan (*SADABS*; Bruker, 2006 ▶) *T*_min_ = 0.978, *T*_max_ = 0.985
11856 measured reflections
1190 independent reflections
1007 reflections with *I* > 2σ(*I*)
*R*_int_ = 0.081

#### Refinement

*R*[*F* ^2^ > 2σ(*F* ^2^)] = 0.046
*wR*(*F* ^2^) = 0.140
*S* = 1.02
1190 reflections
154 parameters
H-atom parameters constrained
Δρ_max_ = 0.19 e Å^−3^
Δρ_min_ = −0.25 e Å^−3^

### 

Data collection: *APEX2* (Bruker, 2006 ▶); cell refinement: *SAINT* (Bruker, 2006 ▶); data reduction: *SAINT*; program(s) used to solve structure: *SHELXS97* (Sheldrick, 2008 ▶); program(s) used to refine structure: *SHELXL97* (Sheldrick, 2008 ▶); molecular graphics: *SHELXTL* (Sheldrick, 2008 ▶); software used to prepare material for publication: *SHELXL97*.

## Supplementary Material

Crystal structure: contains datablocks I, global. DOI: 10.1107/S1600536811009135/rz2565sup1.cif

Structure factors: contains datablocks I. DOI: 10.1107/S1600536811009135/rz2565Isup2.hkl

Additional supplementary materials:  crystallographic information; 3D view; checkCIF report

## Figures and Tables

**Table 1:** Hydrogen-bond geometry (Å, °)

*D*—H⋯*A*	*D*—H	H⋯*A*	*D*⋯*A*	*D*—H⋯*A*
C9—H9*A*⋯O1^i^	0.93	2.57	3.485 (4)	168

Symmetry code: (i) 
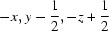
.

**Fig. 1.**
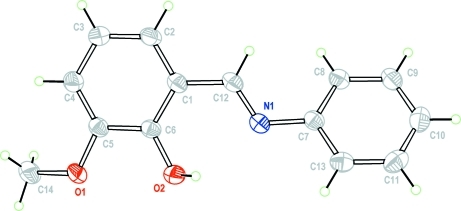
The molecular structure of the title compound. Displacement ellipsoids are drawn at the 30% probability level.

**Fig. 2.**
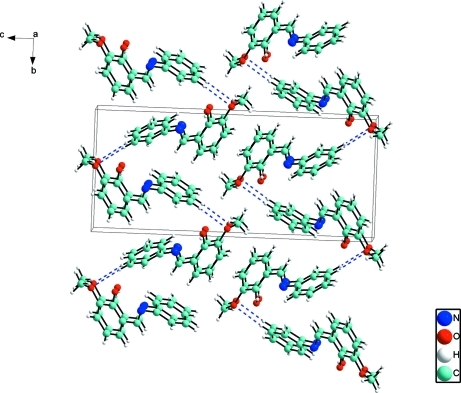
Packing diagram of the title compound compound viewed approximately along the a axis. Intermolecular hydrogen bonds are drawn as dashed lines.

## supplementary crystallographic information

### Comment

For many years, there has been considerable interest in the study of Schiff base compounds due to their biological activity (Zhao *et al.*, 2005). Recently, we have reported the crystal structure of some Schiff bases metal complexes (Zhao *et al.*, 2007; Xian *et al.* 2008; Li *et al.* 2008; Liu *et al.* 2009). As an extention of our work in the structural characterization of Schiff base compounds, we synthesized the title compound and report its crystal structure herein.

In the molecule of the title compound (Fig. 1), the C—O bond lengths range from 1.350 (4) to 1.419 (4) Å. The C—N and C═N bond lengths are 1.428 (3) and 1.285 (4) Å, respectively. The molecule is not planar, with the dihedral angle between the two aromatic rings of 30.57 (10)°. In the crystal structure (Fig. 2), intermolecular C—H···O hydrogen bonds (Table 1) link the molecules into chains parallel to the *b* axis.

### Experimental

Reagents and solvents used were of commercially available quality and were not purified before using. A mixture of *o*-vanillin (0.152 g, 1.0 mmol) and aniline (0.093 g, 1.0 mmol) in absolute ethanol (30 ml) was stirred and refluxed for 4 h. The solution was then cooled to room temperature. Orange single crystals suitable for X-ray diffraction were obtained by slow evaporation of the solvent after about 5 d.

### Refinement

All H atoms were positioned geometrically and refined using a riding model, with C—H = 0.93–0.96 Å, O—H = 0.82 Å, and with *U*_iso_(H) = 1.2*U*_eq_(C, O) or 1.5*U*_eq_(C) for methyl H atoms. In the absence of significant anomalous scattering effects, 820 Friedel pairs were averaged in the final refinement.

### Crystal data

**Table:**

C_14_H_13_NO_2_	*F*(000) = 480
*M**_r_* = 227.25	*D*_x_ = 1.280 Mg m^−^^3^
Orthorhombic, *P*2_1_2_1_2_1_	Mo *K*α radiation, λ = 0.71073 Å
Hall symbol: P 2ac 2ab	Cell parameters from 5745 reflections
*a* = 6.0882 (4) Å	θ = 2.4–25.0°
*b* = 9.1862 (5) Å	µ = 0.09 mm^−^^1^
*c* = 21.0800 (12) Å	*T* = 296 K
*V* = 1178.95 (12) Å^3^	Block, orange
*Z* = 4	0.33 × 0.22 × 0.18 mm

### Data collection

**Table:**

Bruker APEXII area-detector diffractometer	1190 independent reflections
Radiation source: fine-focus sealed tube	1007 reflections with *I* > 2σ(*I*)
graphite	*R*_int_ = 0.081
φ and ω scans	θ_max_ = 25.0°, θ_min_ = 2.4°
Absorption correction: multi-scan (*SADABS*; Bruker, 2006)	*h* = −7→7
*T*_min_ = 0.978, *T*_max_ = 0.985	*k* = −10→10
11856 measured reflections	*l* = −25→23

### Refinement

**Table:**

Refinement on *F*^2^	Primary atom site location: structure-invariant direct methods
Least-squares matrix: full	Secondary atom site location: difference Fourier map
*R*[*F*^2^ > 2σ(*F*^2^)] = 0.046	Hydrogen site location: inferred from neighbouring sites
*wR*(*F*^2^) = 0.140	H-atom parameters constrained
*S* = 1.01	*w* = 1/[σ^2^(*F*_o_^2^) + (0.0787*P*)^2^ + 0.4631*P*] where *P* = (*F*_o_^2^ + 2*F*_c_^2^)/3
1190 reflections	(Δ/σ)_max_ < 0.001
154 parameters	Δρ_max_ = 0.19 e Å^−^^3^
0 restraints	Δρ_min_ = −0.25 e Å^−^^3^

### Special details

**Table:**

Geometry. All esds (except the esd in the dihedral angle between two l.s. planes) are estimated using the full covariance matrix. The cell esds are taken into account individually in the estimation of esds in distances, angles and torsion angles; correlations between esds in cell parameters are only used when they are defined by crystal symmetry. An approximate (isotropic) treatment of cell esds is used for estimating esds involving l.s. planes.
Refinement. Refinement of F^2^ against ALL reflections. The weighted R-factor wR and goodness of fit S are based on F^2^, conventional R-factors R are based on F, with F set to zero for negative F^2^. The threshold expression of F^2^ > 2sigma(F^2^) is used only for calculating R-factors(gt) etc. and is not relevant to the choice of reflections for refinement. R-factors based on F^2^ are statistically about twice as large as those based on F, and R- factors based on ALL data will be even larger.

### Fractional atomic coordinates and isotropic or equivalent isotropic displacement parameters (Å^2^)

**Table:**

	*x*	*y*	*z*	*U*_iso_*/*U*_eq_	
N1	0.1467 (5)	0.8849 (3)	0.19232 (12)	0.0471 (6)	
O2	−0.0125 (4)	1.0344 (2)	0.09751 (11)	0.0546 (6)	
H2B	0.1106	0.9995	0.1017	0.066*	
C6	−0.1608 (5)	0.9260 (3)	0.09144 (13)	0.0425 (7)	
O1	−0.3287 (4)	1.0709 (2)	0.01403 (10)	0.0539 (6)	
C1	−0.1531 (6)	0.7999 (3)	0.12880 (14)	0.0466 (7)	
C5	−0.3315 (6)	0.9420 (3)	0.04705 (14)	0.0454 (7)	
C7	0.2914 (5)	0.8685 (3)	0.24514 (15)	0.0456 (7)	
C10	0.5867 (6)	0.8458 (5)	0.34404 (19)	0.0637 (10)	
H10A	0.6870	0.8378	0.3771	0.076*	
C4	−0.4870 (6)	0.8348 (4)	0.03963 (15)	0.0537 (8)	
H4A	−0.5999	0.8468	0.0104	0.064*	
C12	0.0067 (6)	0.7846 (4)	0.17918 (15)	0.0496 (8)	
H12A	0.0082	0.6993	0.2029	0.060*	
C2	−0.3120 (6)	0.6922 (4)	0.12031 (15)	0.0551 (9)	
H2A	−0.3069	0.6085	0.1451	0.066*	
C8	0.2343 (6)	0.7914 (4)	0.30001 (15)	0.0510 (8)	
H8A	0.0970	0.7477	0.3034	0.061*	
C13	0.4930 (7)	0.9357 (4)	0.24102 (17)	0.0548 (8)	
H13A	0.5290	0.9899	0.2052	0.066*	
C3	−0.4755 (7)	0.7075 (4)	0.07616 (16)	0.0581 (9)	
H3A	−0.5783	0.6338	0.0704	0.070*	
C14	−0.5226 (7)	1.1074 (5)	−0.0198 (2)	0.0693 (11)	
H14A	−0.5026	1.1992	−0.0408	0.104*	
H14B	−0.6436	1.1144	0.0093	0.104*	
H14C	−0.5530	1.0333	−0.0507	0.104*	
C9	0.3840 (6)	0.7807 (4)	0.34918 (17)	0.0578 (9)	
H9A	0.3475	0.7295	0.3858	0.069*	
C11	0.6421 (6)	0.9225 (4)	0.29039 (19)	0.0647 (10)	
H11A	0.7798	0.9655	0.2872	0.078*	

### Atomic displacement parameters (Å^2^)

**Table:**

	*U*^11^	*U*^22^	*U*^33^	*U*^12^	*U*^13^	*U*^23^
N1	0.0523 (14)	0.0443 (13)	0.0446 (14)	0.0027 (14)	0.0003 (13)	0.0060 (11)
O2	0.0561 (13)	0.0449 (13)	0.0629 (14)	−0.0041 (12)	−0.0041 (12)	0.0090 (10)
C6	0.0494 (16)	0.0359 (15)	0.0422 (15)	0.0024 (16)	0.0047 (14)	0.0004 (12)
O1	0.0657 (14)	0.0443 (12)	0.0516 (12)	0.0015 (13)	−0.0101 (12)	0.0121 (10)
C1	0.0581 (18)	0.0405 (16)	0.0413 (15)	0.0056 (17)	0.0016 (15)	0.0024 (13)
C5	0.0549 (17)	0.0401 (16)	0.0413 (15)	0.0055 (16)	0.0021 (15)	0.0010 (13)
C7	0.0501 (16)	0.0387 (16)	0.0479 (17)	0.0054 (15)	0.0012 (14)	0.0004 (13)
C10	0.060 (2)	0.063 (2)	0.068 (2)	0.007 (2)	−0.0147 (19)	−0.009 (2)
C4	0.0639 (19)	0.052 (2)	0.0455 (17)	−0.0025 (18)	−0.0020 (17)	0.0002 (14)
C12	0.0612 (19)	0.0403 (15)	0.0473 (17)	0.0064 (18)	0.0006 (16)	0.0073 (13)
C2	0.075 (2)	0.0416 (17)	0.0491 (17)	−0.008 (2)	−0.0019 (17)	0.0076 (14)
C8	0.0520 (17)	0.0504 (19)	0.0507 (17)	0.0016 (17)	0.0033 (15)	0.0051 (15)
C13	0.0557 (16)	0.0503 (18)	0.058 (2)	−0.0022 (19)	0.0103 (17)	0.0031 (16)
C3	0.066 (2)	0.0484 (18)	0.060 (2)	−0.0104 (19)	−0.0056 (18)	0.0030 (16)
C14	0.069 (2)	0.063 (2)	0.076 (2)	0.007 (2)	−0.014 (2)	0.022 (2)
C9	0.072 (2)	0.050 (2)	0.0511 (18)	0.007 (2)	−0.0057 (19)	0.0037 (15)
C11	0.0486 (18)	0.059 (2)	0.087 (3)	−0.0045 (19)	0.002 (2)	−0.009 (2)

### Geometric parameters (Å, °)

**Table:**

N1—C12	1.285 (4)	C4—C3	1.402 (5)
N1—C7	1.428 (4)	C4—H4A	0.9300
O2—C6	1.350 (4)	C12—H12A	0.9300
O2—H2B	0.8200	C2—C3	1.370 (5)
C6—C1	1.401 (4)	C2—H2A	0.9300
C6—C5	1.406 (4)	C8—C9	1.384 (5)
O1—C5	1.374 (4)	C8—H8A	0.9300
O1—C14	1.419 (4)	C13—C11	1.386 (5)
C1—C2	1.396 (5)	C13—H13A	0.9300
C1—C12	1.447 (5)	C3—H3A	0.9300
C5—C4	1.375 (5)	C14—H14A	0.9600
C7—C13	1.377 (5)	C14—H14B	0.9600
C7—C8	1.400 (5)	C14—H14C	0.9600
C10—C11	1.374 (6)	C9—H9A	0.9300
C10—C9	1.375 (5)	C11—H11A	0.9300
C10—H10A	0.9300		
			
C12—N1—C7	120.1 (3)	C3—C2—C1	121.2 (3)
C6—O2—H2B	109.5	C3—C2—H2A	119.4
O2—C6—C1	122.3 (3)	C1—C2—H2A	119.4
O2—C6—C5	118.7 (3)	C9—C8—C7	119.4 (3)
C1—C6—C5	119.0 (3)	C9—C8—H8A	120.3
C5—O1—C14	116.6 (3)	C7—C8—H8A	120.3
C2—C1—C6	119.4 (3)	C7—C13—C11	119.8 (3)
C2—C1—C12	119.4 (3)	C7—C13—H13A	120.1
C6—C1—C12	121.0 (3)	C11—C13—H13A	120.1
O1—C5—C4	124.6 (3)	C2—C3—C4	119.7 (3)
O1—C5—C6	114.7 (3)	C2—C3—H3A	120.1
C4—C5—C6	120.7 (3)	C4—C3—H3A	120.1
C13—C7—C8	120.0 (3)	O1—C14—H14A	109.5
C13—C7—N1	117.0 (3)	O1—C14—H14B	109.5
C8—C7—N1	123.0 (3)	H14A—C14—H14B	109.5
C11—C10—C9	120.5 (4)	O1—C14—H14C	109.5
C11—C10—H10A	119.7	H14A—C14—H14C	109.5
C9—C10—H10A	119.7	H14B—C14—H14C	109.5
C5—C4—C3	120.0 (3)	C10—C9—C8	120.1 (3)
C5—C4—H4A	120.0	C10—C9—H9A	120.0
C3—C4—H4A	120.0	C8—C9—H9A	120.0
N1—C12—C1	122.3 (3)	C10—C11—C13	120.2 (3)
N1—C12—H12A	118.8	C10—C11—H11A	119.9
C1—C12—H12A	118.8	C13—C11—H11A	119.9

### Hydrogen-bond geometry (Å, °)

**Table:**

*D*—H···*A*	*D*—H	H···*A*	*D*···*A*	*D*—H···*A*
C9—H9A···O1^i^	0.93	2.57	3.485 (4)	168

Symmetry codes: (i) −*x*, *y*−1/2, −*z*+1/2.
